# Non-linear Heart Rate and Blood Pressure Interaction in Response to Lower-Body Negative Pressure

**DOI:** 10.3389/fphys.2017.00767

**Published:** 2017-10-24

**Authors:** Ajay K. Verma, Da Xu, Amanmeet Garg, Anita T. Cote, Nandu Goswami, Andrew P. Blaber, Kouhyar Tavakolian

**Affiliations:** ^1^Department of Electrical Engineering, University of North Dakota, Grand Forks, ND, United States; ^2^Department of Biomedical Physiology and Kinesiology, Simon Fraser University, Burnaby, BC, Canada; ^3^Department of Engineering Science, Simon Fraser University, Burnaby, BC, Canada; ^4^School of Human Kinetics, Trinity Western University, Langley, BC, Canada; ^5^Institute of Physiology, Medical University of Graz, Graz, Austria

**Keywords:** hemorrhage, causality, baroreflex, heart rate, arterial blood pressure, central hypovolemia, blood loss

## Abstract

Early detection of hemorrhage remains an open problem. In this regard, blood pressure has been an ineffective measure of blood loss due to numerous compensatory mechanisms sustaining arterial blood pressure homeostasis. Here, we investigate the feasibility of causality detection in the heart rate and blood pressure interaction, a closed-loop control system, for early detection of hemorrhage. The hemorrhage was simulated via graded lower-body negative pressure (LBNP) from 0 to −40 mmHg. The research hypothesis was that a significant elevation of causal control in the direction of blood pressure to heart rate (i.e., baroreflex response) is an early indicator of central hypovolemia. Five minutes of continuous blood pressure and electrocardiogram (ECG) signals were acquired simultaneously from young, healthy participants (27 ± 1 years, *N* = 27) during each LBNP stage, from which heart rate (represented by RR interval), systolic blood pressure (SBP), diastolic blood pressure (DBP), and mean arterial pressure (MAP) were derived. The heart rate and blood pressure causal interaction (RR↔SBP and RR↔MAP) was studied during the last 3 min of each LBNP stage. At supine rest, the non-baroreflex arm (RR→SBP and RR→MAP) showed a significantly (*p* < 0.001) higher causal drive toward blood pressure regulation compared to the baroreflex arm (SBP→RR and MAP→RR). In response to moderate category hemorrhage (−30 mmHg LBNP), no change was observed in the traditional marker of blood loss i.e., pulse pressure (*p* = 0.10) along with the RR→SBP (*p* = 0.76), RR→MAP (*p* = 0.60), and SBP→RR (*p* = 0.07) causality compared to the resting stage. Contrarily, a significant elevation in the MAP→RR (*p* = 0.004) causality was observed. In accordance with our hypothesis, the outcomes of the research underscored the potential of compensatory baroreflex arm (MAP→RR) of the heart rate and blood pressure interaction toward differentiating a simulated moderate category hemorrhage from the resting stage. Therefore, monitoring baroreflex causality can have a clinical utility in making triage decisions to impede hemorrhage progression.

## Introduction

Hemorrhage, a physiological condition resulting in central hypovolemia; owing to loss of blood from the circulation which leads to inadequate tissue perfusion (Schiller et al., 2017), is a major cause of death in soldiers and civilians following a trauma (Søreide et al., 2007; Evans et al., 2010; Eastridge et al., 2012; Rhee et al., 2014). Additionally, postpartum hemorrhage is a recognized cause of maternal mortality (Hancock et al., 2015), especially in low-income countries (Say et al., 2014). The capability to track hemorrhage progression early, accordingly, can be indispensable toward impeding hemorrhage advancement via appropriate intervention (Beekley et al., 2008; Gerhardt et al., 2011). Reliance on arterial blood pressure as an early indicator of central blood loss has been ineffectual, due to various compensatory mechanisms maintaining arterial blood pressure homeostasis until the point of autonomic decompensation (Soller et al., 2008; Convertino et al., 2015). Blood pressure falls abruptly upon autonomic decompensation, followed by systemic hypotension, which, if not immediately compensated for, could result in tissue hypoperfusion (hemorrhagic shock; Victorino et al., 2003; Parks et al., 2006; Schiller et al., 2017).

Under the physiological state of systemic hypotension (systolic blood pressure <90 mmHg), the interventional strategies have limited effect (Gerhardt et al., 2011; Tavakolian et al., 2014). Pulse pressure and heart rate are recommended or considered for triage decisions (Convertino et al., 2006; Brasel et al., 2007), however, pertinent literature reveals heart rate alone is an unreliable marker of blood loss due its dependency on numerous factors such as, pain, vagal and sympathetic tone, and hormones, as well as showing late changes in response to central hypovolemia (Victorino et al., 2003; Cooke et al., 2006; Brasel et al., 2007; Soller et al., 2008), while high inter-subject variability has been demonstrated with pulse pressure as a marker of central blood loss (Cote et al., 2012). The quantification of compensatory mechanisms, corresponding to the maintenance of blood pressure homeostasis, therefore, could dispense paramount information pertaining to the progression of central hypovolemia (Nadler et al., 2014; Convertino et al., 2015, 2016; Janak et al., 2015).

Baroreceptors, the stretch receptors localized in the carotid sinus and the aortic arch play a central role toward maintenance of blood pressure homeostasis (Heesch, 1999). The perturbation to circulatory homeostasis via redistribution of central blood volume away from the heart and the associated decline in the arterial blood pressure is sensed by the stretch receptors on the blood vessels, which leads to a reduction in baroreceptor discharge to the brain. Rapid withdrawal of vagal and activation of sympathetic nerve activity, which causes an elevation in systemic vascular resistance (SVR) and heart rate, is a consequent baroreceptor mediated reflex response to an external perturbation to regulate arterial blood pressure (Rowell, 1993; Ricci et al., 2015). The baroreflex activity quantified via muscle sympathetic nerve activity has been shown to track central hypovolemia (Cooke et al., 2009; Ryan et al., 2012), however, given the requirement of sophisticated instrumentation and invasive nature; it has limited application toward surgical triage. The non-invasive quantification of sympathetic tone via heart rate variability has also been explored to track central hypovolemia, nonetheless, high inter-subject variability has been reported with such an approach (Cooke et al., 2006; Ryan et al., 2010).

In response to an external perturbation to the hemodynamic homeostasis, heart rate, and blood pressure are known to act in conjunction (closed loop) toward maintenance of arterial blood pressure homeostasis (Porta et al., 2002, 2011; Faes et al., 2013). This causal interaction has feedforward (non-baroreflex) and feedback (baroreflex) controls, governed by the Frank-Starling mechanism on blood pressure and the baroreflex mediated control of heart rate, respectively (Porta et al., 2011; Faes et al., 2013; Javorka et al., 2017). The imperative role of causal heart rate and blood pressure interaction toward regulation of arterial blood pressure is well documented in the literature under external disruption to the hemodynamic homeostasis, induced by head-up tilt and stand tests (Nollo et al., 2005; Porta et al., 2011, 2017; Javorka et al., 2017). The physiological response of the cardiovascular system to such stressors can be hypothesized to be analogous to blood loss simulated via the lower-body negative pressure (LBNP), as all experimental protocols aim to translocate central blood volume downwards to the peripheral regions, as a consequence of gravity in a head-up tilt or stand test and lower body suction in the LBNP protocol.

LBNP is an acclaimed experimental tool for replicating controlled central hypovolemia of variable degree in a laboratory setting. The response of the cardiovascular system to LBNP has been shown to be analogous to hemorrhage (Cooke et al., 2004; Blaber et al., 2013; Hinojosa-Laborde et al., 2014b; Johnson et al., 2014; Janak et al., 2015). Therefore, LBNP provides a practical, reproducible, and a safe physiological surrogate to study the hemodynamic response manifesting hemorrhage (Cooke et al., 2004). The continuous application of LBNP sequesters central blood volume in the compliant venous system of lower peripheral regions (Goswami et al., 2008; Blaber et al., 2013), causing a substantial decline in the central venous return and preload. Consequently, a reduction in stroke volume and cardiac output is observed (Cooke et al., 2004; Blaber et al., 2013; Hinojosa-Laborde et al., 2014b).

Depending on the amount of blood volume lost from the circulation, a hemorrhage is categorized as a mild, moderate, or severe (Cooke et al., 2004). The LBNP stage of −20 to −40 mmHg simulates the spectrum of a moderate category hemorrhage, displacing 10–20% of total blood volume or approximately up to 1,000 ml of blood loss (Cooke et al., 2004). Therefore, a successful differentiation of compensatory mechanisms regulating arterial blood pressure at −30 mmHg LBNP from rest can serve as a potential tool toward effective triage, to impede hemorrhage advancement to a severe category.

In the current research, we systematically studied the causal behavior of the heart rate and blood pressure interaction at rest and under graded LBNP to −40 mmHg. Since LBNP is known to reduce stroke volume (Cooke et al., 2004; Hinojosa-Laborde et al., 2014b; Tavakolian et al., 2014), the directional behavior was studied with respect to LBNP, to gain inference regarding the dynamics of closed loop heart rate and blood pressure interaction in response to simulated blood loss. The interaction was quantified using a non-linear convergent cross mapping (CCM) method. The potential of CCM method in outlining the directional information flow between physiological signals has been demonstrated in the literature (Heskamp et al., 2014; Schiecke et al., 2015, 2016; Verma et al., 2016, 2017; Xu et al., 2017). Additionally, given the inherent non-linear nature of heart rate and blood pressure signals, the CCM method holds advantage toward unraveling underlying directional information flow compared to traditional Granger causality, which has been limited in effect with signals of non-linear nature (Schiecke et al., 2016).

The non-linear causal relationship between heart rate and blood pressure in response to LBNP induced central hypovolemia remains to be generalized. The current work is an attempt toward studying this relationship in response to simulated hemorrhage with a hypothesis that such non-linear relationship can assist early detection of blood loss. As LBNP decreases venous return, and heart rate and blood pressure show causal interaction, we hypothesize a significant contribution from the feedback arm of such an interaction; that is, the baroreflex mediated heart rate control in response to LBNP.

## Methods

### Experimental protocol

The lower body of each participant was placed in the LBNP chamber and sealed at the level of the iliac crest. The participants lay supine inside the chamber for 5 min after which, the pressure inside the chamber was gradually reduced to −20 mmHg, from this point the chamber pressure was reduced in steps of 10 mmHg up to −40 mmHg. Five minutes of negative pressure was applied at each LBNP stage. A straddling bicycle seat inside the chamber prevented participants from getting further pulled inside the chamber. The chamber pressure was immediately terminated if a participant exhibited (1) pre-syncopal symptoms, (2) sudden drop in blood pressure and/or heart rate (3) any discomfort, or (4) upon request.

The experimentation was conducted in the Aerospace Physiology Laboratory in the Department of Biomedical Physiology and Kinesiology, Simon Fraser University (SFU), Canada. A participant in the age range of 18–40 years and without a history of cardiovascular disease was eligible to participate in the study. The experimental protocol was approved to be of minimal risk and complied with the rules and regulations set forth by the research ethics board of SFU. Written informed consent for participation was obtained from each participant prior to any experimentation. A registered nurse was present during experimentation for the safety of participants.

### Data acquisition

Simultaneous electrocardiogram (ECG) and blood pressure were acquired from 27 young, healthy participants (15 males and 12 females, age: 27 ± 1 years, weight: 66 ± 2 kg, height: 169 ± 2 cm, mean ± SE) who underwent graded LBNP. The ECG signal was acquired in a lead II configuration using LifePak8 (Medtronic Inc., MN, USA) and the blood pressure signal was acquired using a finger photoplethysmograph cuff (FMS, Amsterdam, The Netherlands) applied on the mid phalanx of the middle finger (left hand). Five minutes of data were acquired during rest i.e., baseline and each LBNP stages using an NI 9205 analog input module (National Instruments Inc., TX, USA) at a sampling rate of 1,000 Hz.

### Convergent cross mapping

CCM proposed by Sugihara et al. is a non-linear approach of studying cause and effect relationship based on the state space reconstruction of a time series (Sugihara et al., 2012). The causality between two time series is inferred by studying the correspondence between the manifolds constructed using the optimal dimension of reconstruction and a delay (Krakovská et al., 2015; Ye et al., 2015). The history of response is used to estimate the states of the driver, contrary to the concept of causality proposed by Granger (Schiecke et al., 2015). If there exists a causal information flowing from X to Y (X→Y), then the states of X can be successfully estimated using the states of Y (Sugihara et al., 2012; McCracken, 2016). The strength of this causality is quantified by computing Pearson correlation coefficient between estimated and original variable. The causal information flowing from Y to X is represented as *Y* → *X* = ρ(*Y*, *Ŷ*|*M*_*X*_), similarly, the causal information flowing from X to Y is represented as $X\to Y=\rho \left(X,\;\hat{X}|M_{Y}\right)$.

Where *M*_*X*_ and *M*_*Y*_ represent the manifold of X and Y, respectively constructed using time delay and dimension of reconstruction, $\hat{X}|M_{Y}$ and *Ŷ*|*M*_*X*_ are estimates of variable X (using Y manifold) and Y (using X manifold), respectively, and ρ is the Pearson correlation coefficient signifying the strength of coupling. Further details of the methodology to infer a causal relationship between two time series is presented in the supplementary material of Sugihara et al. (2012) and in a book on time series analysis by McCracken (2016).

### Data processing

From the acquired signals, beat-to-beat R-R intervals, SBP, and diastolic blood pressure (DBP) were obtained using Beatscope software (Finapres, FMS, The Netherlands). The mean arterial pressure (MAP) was obtained from the systolic and DBP as; $MAP=\frac{2}{3}\times DBP+\frac{1}{3}\times SBP$. An evenly sampled signal was generated using spline interpolation from beat-to-beat signals and was resampled to 10 Hz prior to causality analysis. Only the last 3 min from each LBNP stage was considered for analysis to allow for effective blood pooling induced by LBNP and its effect on the cardiovascular parameters. The optimal dimension of reconstruction (*M*) was determined according to the false nearest neighbor algorithm in MATLAB using CRP toolbox (Kennel and Brown, 1992; Marwan, 2017), while the delay (τ) was chosen to be 10 samples to account for changes within a heartbeat range. The false nearest neighbor minimization of approximately 95% was achieved for the last 3 min of SBP, MAP, and RR at *M* = 3. To limit the effect of noise, which may otherwise lead to a determination of higher dimension of reconstruction, all causality values reported were performed at *M* = 3 and τ = 10. For each stage, the correlation coefficient value at which each causal event (i.e., SBP→RR, MAP→RR, RR→SBP, and RR→MAP) converged was considered as a degree of directional information flow from one variable to another. The significance of correlation was set at α = 0.05. The MATLAB (Mathworks Inc., MA) implementation of CCM algorithm demonstrated in an application with non-linear signals in the study conducted by Krakovská et al. (2015) was considered for analysis.

### Statistical analysis

The group mean of RR, SBP, DBP, MAP, pulse pressure (PP = SBP-DBP), the non-baroreflex i.e., feedforward (RR→SBP and RR→MAP), and the baroreflex i.e., feedback (SBP→RR and MAP→RR) causality values for the last 3 min of each LBNP stage was obtained. Test for normality of the data was conducted using the Shapiro-Wilk test at α = 0.05. A one-way test of ANOVA (normally distributed data) or Wilcoxon rank sum test (data failed the normality test) was conducted to test the significance of the difference. The group mean of baroreflex causality (SBP→RR and MAP→RR) was compared with the group mean of non-baroreflex causality (RR→SBP and RR→MAP) under baseline using one-way ANOVA. A multiple comparison test, to account for the significance of the difference in the cardiovascular parameters and the SBP↔RR and MAP↔RR causality, inflicted by different LBNP stages, was conducted using one-way ANOVA followed by *post-hoc* analysis using the Tukey-HSD method. All tests for significance were conducted using a statistical toolbox of MATLAB (Mathworks Inc., MA, USA). The test result at α = 0.05 was considered as significant. All tabular results are presented as mean ± SD while all graphical results are presented as mean ± SE unless mentioned otherwise.

## Results

**Figure 1:**
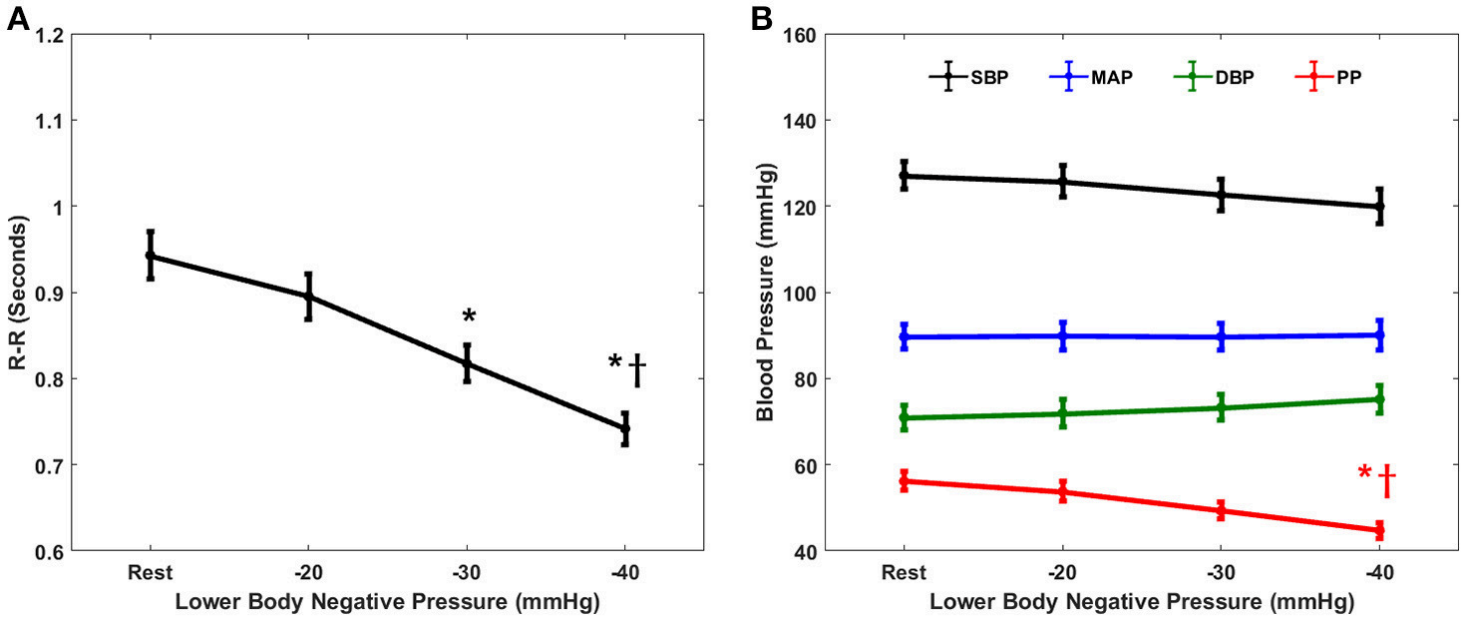
The response of cardiovascular parameters to graded lower-body negative pressure. R-R intervals **(A)** reduced significantly both at −30 (*p* = 0.001) and −40 (*p* < 0.001) mmHg LBNP compared to rest. Pulse pressure **(B)** reduced significantly (*p* = 0.001) at −40 mmHg compared to rest. Additionally, both R-R intervals (*p* < 0.001) and pulse pressure (*p* = 0.02) reduced significantly at −40 mmHg LBNP compared to −20 mmHg LBNP. The systolic blood pressure **(B)**, diastolic blood pressure **(B)**, and the mean arterial pressure **(B)** did not change (*p* = 0.50, *p* = 0.79, and *p* = 0.99, respectively) in response to graded lower-body negative pressure, ^*^ and ^†^represents significant change (*p* < 0.05, *post-hoc* result) compared to rest and −20 mmHg, respectively.

The cardiovascular parameters (RR, SBP, DBP, MAP, PP), as well as the non-baroreflex and the baroreflex causal events, passed the test of normality (*p* > 0.05). The behavior of cardiovascular parameters with progressive LBNP is summarized in Figure 1. The effect of different LBNP stages on the cardiovascular parameters was compared using one-way ANOVA followed by *post-hoc* analysis using the Tukey-HSD method, the resulting *p*-value of the comparison is summarized in Table 1. A significant reduction in pulse pressure (*p* = 0.001) at −40 mmHg, and RR interval at −30 mmHg (*p* = 0.001), and −40 mmHg (*p* < 0.001) was observed compared to the resting stage. Additionally, a significant difference was observed between −20 and −40 mmHg LBNP in PP (*p* = 0.02) and RR interval (*p* < 0.001), Figure 1. In response to different LBNP stages, we observed no change in SBP (*p* = 0.50), DBP (*p* = 0.79), and MAP (*p* = 0.99), Figure 1.

**Table 1:** Comparison of the response of variables to different LBNP stages.

**LBNP Stages for Comparison**		**R-R**	**PP**	**SBP→RR**	**MAP→RR**
Rest	−20 mmHg	0.49	0.85	0.74	0.50
Rest	−30 mmHg	0.001^*^	0.10	0.07	0.004^*^
Rest	−40 mmHg	<0.001^*^	0.001^*^	0.001^*^	<0.001^*^
−20 mmHg	−30 mmHg	0.10	0.44	0.46	0.19
−20 mmHg	−40 mmHg	<0.001^*^	0.02^*^	0.04^*^	0.01^*^
−30 mmHg	−40 mmHg	0.12	0.42	0.60	0.72

*The table lists post-hoc analysis (Tukey-HSD method) p-value for different comparisons*.

* *Represents significant difference (p < 0.05). Only the variables with significant (p < 0.05) one-way ANOVA results are listed*.

*R-R, RR intervals; PP, Pulse pressure; SBP→RR, systolic blood pressure to heart rate causality (baroreflex); MAP→RR, mean arterial pressure to heart rate causality (baroreflex); LBNP, Lower-body negative pressure*.

**Figure 2:**
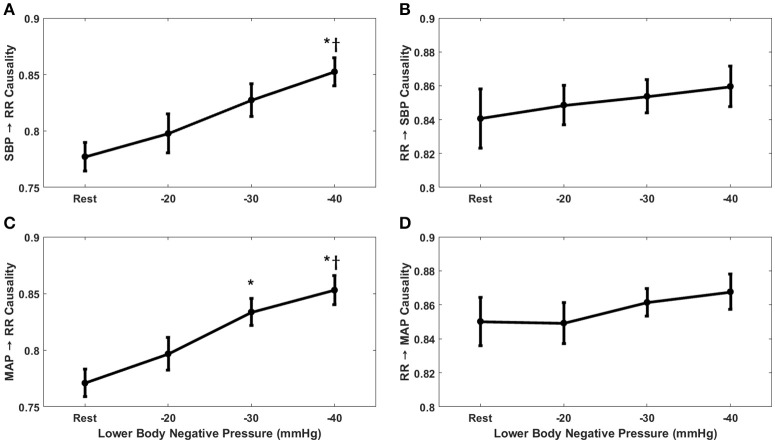
The response of baroreflex **(A,C)** and non-baroreflex **(B,D)** causalities to graded lower-body negative pressure. In response to LBNP, MAP→RR (*p* = 0.004) causality increased significantly at −30 mmHg compared to rest, SBP→RR (*p* = 0.001) causality increased significantly at −40 mmHg compared to rest. Compared to −20 mmHg the SBP→RR (*p* = 0.04) and MAP→RR (*p* = 0.01) causality increased significantly at −40 mmHg LBNP. No change in RR→SBP (*p* = 0.76) and RR→MAP (*p* = 0.60) causality was observed in response to LBNP. ^*^Represents significant difference (*p* < 0.05, *post-hoc* result) from rest while ^†^represents significant difference from −20 mmHg.

**Figure 3:**
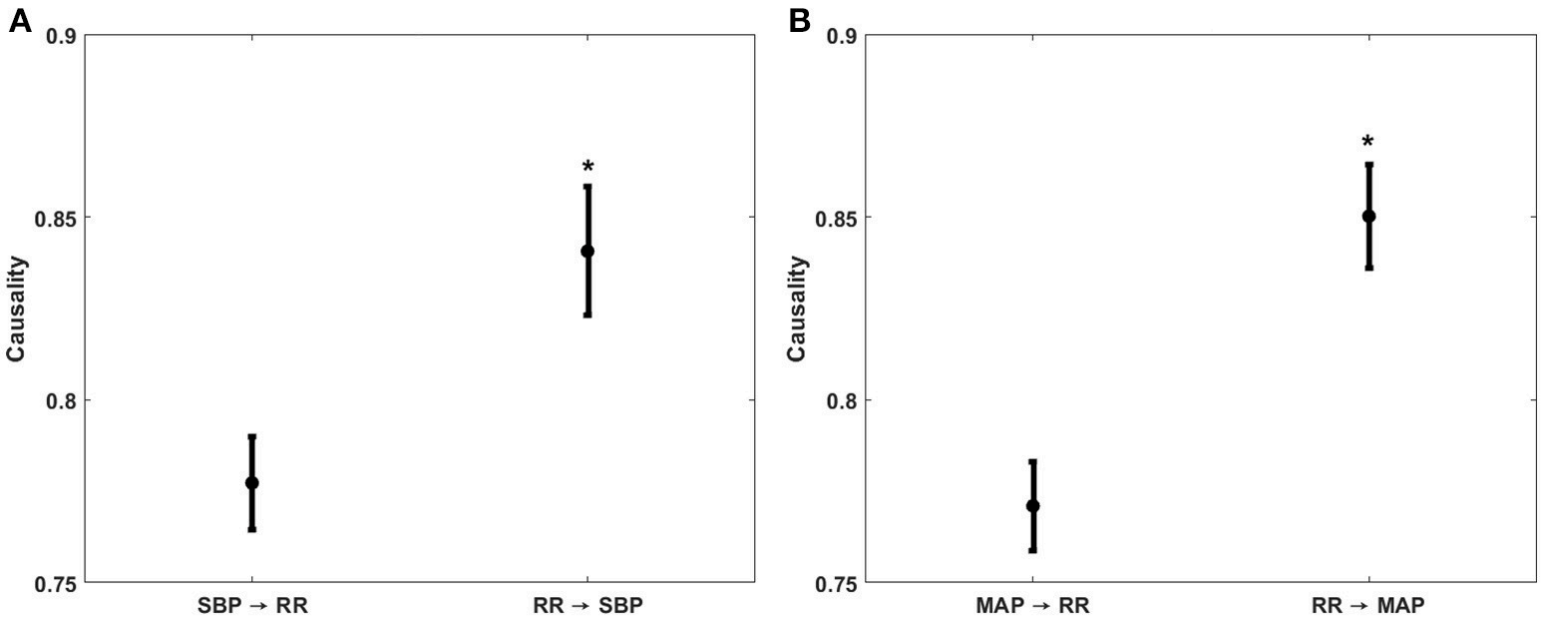
Comparison of non-baroreflex or feedforward (RR→SBP and RR→MAP) and baroreflex or feedback (SBP→RR and MAP→RR) causalities during supine baseline. The feedforward causality was significantly stronger (*p* < 0.001) than the feedback causality for both RR↔SBP **(A)** and RR↔MAP **(B)** interactions. ^*^Represents significantly (*p* < 0.05, one-way ANOVA) stronger causality.

The compensatory behavior, responsible for maintaining blood pressure equilibrium via SBP↔RR and MAP↔RR interaction was quantified in response to progressing LBNP. This behavior is summarized in Figure 2. During the resting stage, a significantly stronger causal drive from heart rate toward SBP (RR→SBP) and MAP (RR→MAP) in comparison to causal drive in the reverse direction (SBP→RR and MAP→RR; Figure 3) was observed. With the increase in the lower-body negative pressurization, the causal strength of the baroreflex arm (MAP→RR) increased and achieved a statistical significance as early as −30 mmHg LBNP compared to rest while the baroreflex causality via SBP→RR interaction did not change until −40 mmHg LBNP (Figure 2). In response to LBNP, the RR→SBP (*p* = 0.76) and RR→MAP (*p* = 0.60) causality did not change (Figure 2). The SBP→RR (*p* = 0.04) and MAP→RR (*p* = 0.01) successfully differentiated −40 mmHg LBNP from −20 mmHg LBNP. Table 2 lists the value of all variables studied in this research (mean ± SD) in response to LBNP.

**Table 2:** Response of different variables to graded lower-body negative pressure.

**Variables vs. LBNP**	**Rest**	**−20 mmHg**	**−30 mmHg**	**−40 mmHg**
R-R (s)	0.94 ± 0.14	0.90 ± 0.14	0.82 ± 0.11^*^	0.74 ± 0.10^*^^†^
SBP (mmHg)	127.1 ± 16.45	125.7 ± 18.90	122.6 ± 18.59	119.9 ± 21.26
DBP (mmHg)	70.9 ± 15.17	71.9 ± 16.50	73.3 ± 15.62	75.2 ± 16.54
MAP (mmHg)	89.7 ± 14.65	89.9 ± 16.39	89.7 ± 15.95	90.1 ± 17.66
PP (mmHg)	56.2 ± 11.40	53.8 ± 11.98	49.3 ± 10.28	44.7 ± 9.80^*^^†^
RR→SBP	0.84 ± 0.09	0.85 ± 0.06	0.85 ± 0.05	0.86 ± 0.06
SBP→RR	0.78 ± 0.07	0.80 ± 0.09	0.83 ± 0.07	0.85 ± 0.07^*^^†^
RR→MAP	0.85 ± 0.07	0.85 ± 0.06	0.86 ± 0.04	0.87 ± 0.05
MAP→RR	0.77 ± 0.06	0.80 ± 0.07	0.83 ± 0.06^*^	0.85 ± 0.07^*^^†^

*The table lists the value of each variable for respective LBNP stages*.

* Represents significant (Tukey-HSD post-hoc analysis, p < 0.05) difference compared to rest and

† *represents significant difference compared to −20 mmHg*.

*LBNP, Lower-body negative pressure; R-R, RR intervals; SBP, systolic blood pressure; DBP, diastolic blood pressure; MAP, mean arterial pressure; PP, pulse pressure; RR→SBP, heart rate to SBP causality (non-baroreflex); SBP→RR, SBP to heart rate causality (baroreflex); RR→MAP, heart rate to MAP causality (non-baroreflex); and MAP→RR, MAP to heart rate causality (baroreflex)*.

**Figure 4:**
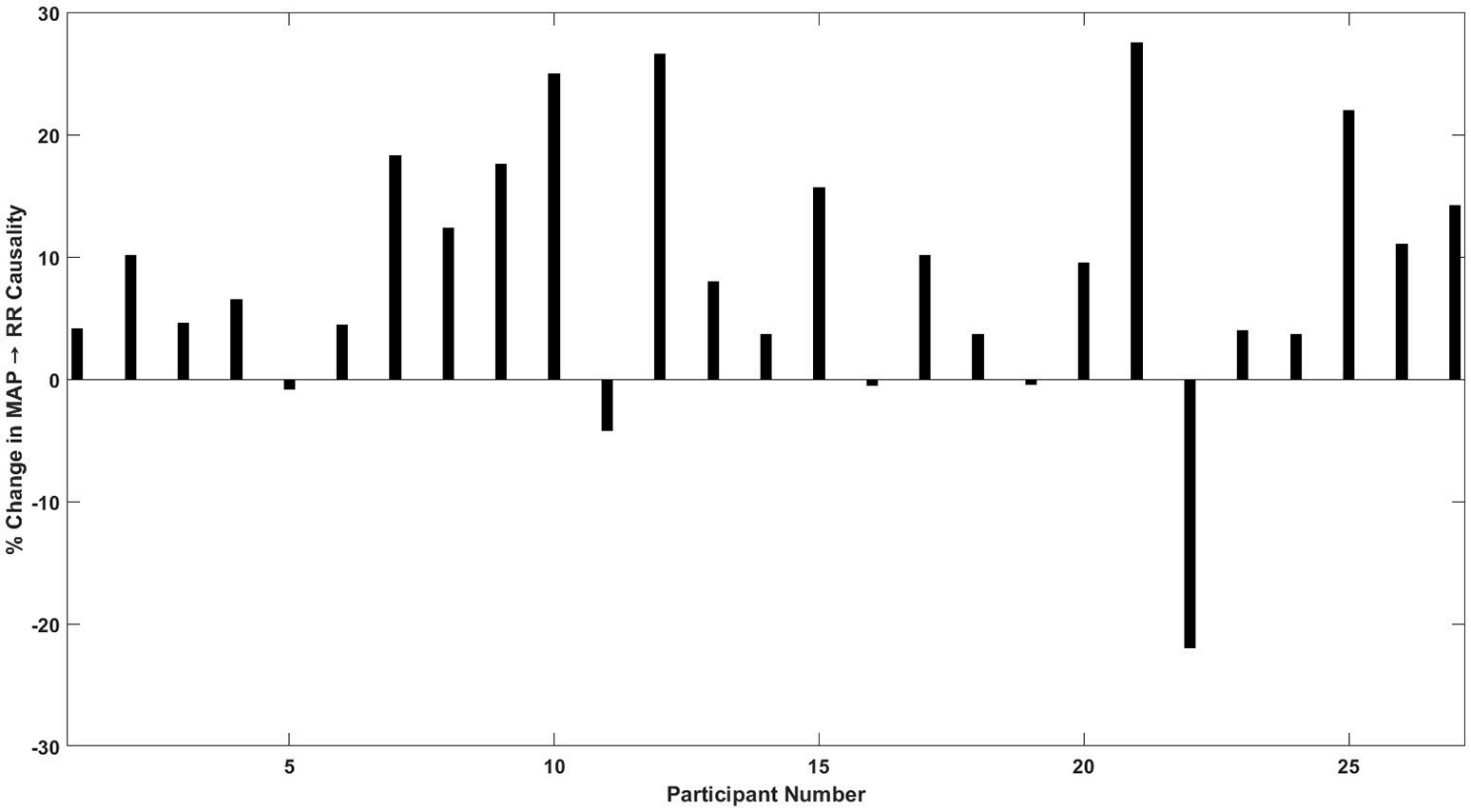
Percent (%) change in MAP→RR causality in response to −30 mmHg LBNP compared to rest. Out of 27 participants, 22 showed an increase, 2 participants showed a decline, and 3 participants did not show a change in MAP→RR causality at moderate LBNP compared to rest.

Figure 4 details the percent change in baroreflex causality (MAP→RR) at −30 mmHg LBNP compared to resting stage for individual study participants. Eighty two percentage participants showed an increase in MAP→RR causality during −30 mmHg LBNP compared to baseline. The group mean of baroreflex causalities (SBP→RR and MAP→RR), RR, and PP other than able to differentiate moderate category (−30 or −40 mmHg LBNP) hemorrhage from resting baseline, showed a significant correlation (*p* < 0.05) with the LBNP stages. Also, the group mean of SBP, DBP, and RR→SBP was significantly (*p* < 0.05) correlated with LBNP stages while the group mean of MAP (*p* = 0.26) and RR→MAP (*p* = 0.14) showed no relation with the LBNP stages.

## Discussion

The major finding of the current research was the feasibility of the causal heart rate and blood pressure interaction toward differentiating the onset of moderate intensity of simulated hemorrhage from the resting stage. A significant elevation was observed in the strength of baroreflex arm (MAP→RR) of the heart rate blood pressure interaction in response to the LBNP induced redistribution of central blood volume to the lower peripheral parts. This elevation was evident as early as −30 mmHg of LBNP. On the contrary, the conventional indicators of blood loss i.e., blood pressure (SBP, DBP, MAP, or PP) failed to differentiate −30 mmHg LBNP intensity from the resting stage, thus, highlighting the potential of compensatory baroreflex arm of the heart rate and blood pressure interaction toward early detection of progressing blood loss.

Early detection of hemorrhage progression has eluded the research and clinical communities for years. Stroke volume is shown to be an early indicator of mild hypovolemia or blood loss (Westphal et al., 2007; Elstad et al., 2009; Holme et al., 2016), however, an accurate measurement of stroke volume entails sophisticated instrumentation and an expert operator (Scherhag et al., 2005), thus, limiting its application in the environment where majority of hemorrhagic shock occurs. Therefore, non-invasively acquired arterial blood pressure is often relied on for surgical triage. Nevertheless, numerous physiological mechanisms responsible for arterial blood pressure regulation have limited the efficacy of blood pressure from exhibiting early symptoms of blood loss from the circulation. To limit mortality, corresponding to hemorrhagic shock via effective triage, there is a profound interest toward early detection of hemorrhage non-invasively. Pulse pressure has been proposed in the literature to obtain early insights regarding hemorrhage progression, for its association with stroke volume (Convertino et al., 2006). In an alignment with existing findings in the literature, the current research found a strong and significant correlation between PP and LBNP stages. However, PP (*p* = 0.10) failed to show a significant change in its dynamics compared to resting stage until the tail end of moderate category hemorrhage (−40 mmHg), which raises concern regarding its potential to provide an early trace of central blood loss.

Furthermore, cardiovascular parameters such as, SBP and DBP were significantly (*p* < 0.05) correlated with LBNP stages, however, neither of them showed a significant change in their dynamics with the application of LBNP simulating moderate category hemorrhage (Figure 1). This observation further highlighted the ineffectiveness of the cardiovascular parameters alone as an early indicator of blood loss. Out of all cardiovascular parameters studied in this research, we found heart rate to be most sensitive to LBNP, showing significant change (*p* = 0.001) as early as −30 mmHg compared to resting baseline (Figure 1). However, making a triage decision based on heart rate alone has been a subject of controversy due to the association of heart rate with several physiological mechanisms (Victorino et al., 2003; Brasel et al., 2007) as well as owing to existing discrepancies in the literature whether heart rate is an early or late marker of central hypovolemia (Convertino et al., 2006, 2016; Cooke et al., 2006; Soller et al., 2008). In addition to heart rate, pulse pressure was observed to be superior to SBP, MAP, and DBP when tracking simulated blood loss via LBNP. Even though PP failed to differentiate −30 mmHg from baseline, it successfully differentiated tail end of moderate category hemorrhage (−40 mmHg) from resting stage and −40 mmHg LBNP from −20 mmHg LBNP, while SBP, DBP, and MAP showed no change with the application of LBNP (Figure 1).

To impede progressing hemorrhage, it is central to quantify the compensatory mechanisms regulating arterial blood pressure. To this end, the current research investigated the sensitivity of causal heart rate and blood pressure interaction for monitoring simulated central hypovolemia. Furthermore, owing to the robustness of the non-linear methodology, we successfully highlighted the contribution of the non-baroreflex (RR→SBP and RR→MAP) and the baroreflex (SBP→RR and MAP→RR) mechanisms responsible for blood pressure regulation under a variable degree of LBNP induced physiological stressor (Figure 2). The directional information flow, mediated by both baroreflex and non-baroreflex arms of the interaction was observed, with the non-baroreflex (RR→SBP and RR→MAP) arm being dominant (*p* < 0.001) during the resting stage compared to the baroreflex arm (SBP→RR and MAP→RR), Figure 3.

With the application of external perturbation to the hemodynamic homeostasis in the form of lower body negative pressure, an elevation in the causal activity, in the direction of blood pressure to heart rate (SBP→RR and MAP→RR) was observed, representing activation of baroreflex mediated control of heart rate toward maintenance of arterial blood pressure homeostasis. Progression of central hypovolemia to moderate intensity (−30 mmHg) was accompanied by no significant elevation in the SBP→RR (*p* = 0.07) causality but significant elevation in the causal drive from MAP→RR (*p* = 0.004) while no change was observed in the strength of reverse drive, which is a representative of heart rate mediated blood pressure changes i.e., RR→SBP (*p* = 0.76) and RR→MAP (*p* = 0.60). This observation indicated that under resting condition blood pressure is primarily maintained through blood pressure changes mediated by heart rate, contrarily, under physiologically perturbed cardiovascular system due to a decline in venous return, the baroreflex mediated heart rate control acts as a compensatory mechanism leading to arterial blood pressure homeostasis. Thus, the two blood pressure regulatory mechanisms interact in closed loop at any given time in order to maintain blood pressure homeostasis. Furthermore, the baroreflex causality (SBP→RR and MAP→RR) was able to differentiate −40 mmHg from −20 mmHg, therefore highlighting its capability to track and differentiate varying intensity of hemorrhage (Table 1).

This behavior of closed loop heart rate and blood pressure interaction under varying physiological conditions ascertained the contribution of either arm of the blood pressure regulation mechanism. The findings of the current research corroborated with the previous findings regarding heart rate and blood pressure interaction highlighted under head-up tilt and the stand test, which demonstrated an elevation in the baroreflex activity during orthostatic challenge compared to baseline with SBP being an indicator of blood pressure functioning (Czippelova et al., 2014; Javorka et al., 2017; Silvani et al., 2017). However, the literature is limited in terms of comprehensive quantified knowledge of such non-linear behavior with respect to progressing blood loss simulated by LBNP.

The report by Dorantes-Mendez et al. (2013) and Silvani et al. (2017) investigated heart rate and blood pressure coupling with respect to LBNP and actual blood loss, respectively. However, a linear methodological approach was considered for such quantification. Moreover, the heart rate and blood pressure interaction is known to be of non-linear nature, therefore, a more robust approach would be a prerequisite, for accurately underpinning the continuous dynamics of the non-baroreflex and the baroreflex arms of such interaction. With the application of non-linear methodology and higher sample size compared to previous two works, in the current research, we systematically demonstrated the degree of statistical alteration in both the non-baroreflex and the baroreflex mechanisms of blood pressure regulation in response to the simulated progressing hemorrhage (LBNP). Our study, therefore, provided comprehensive insights regarding the feasibility of compensatory directional interaction for monitoring progression of hemorrhage and for surgical triage.

Additionally, we compared the use of SBP and MAP as a marker of baroreflex mediated heart rate control (baroreflex causality) in response to LBNP. As such, we found MAP to be a more sensitive marker of baroreflex mediated heart rate control in response to moderate category (−30 mmHg) LBNP compared to SBP (Figure 2). The MAP→RR causality achieved statistical significance at −30 mmHg LBNP compared to baseline while the SBP→RR causality did not show a significant change in its dynamics until −40 mmHg LBNP. However, SBP→RR and MAP→RR both showed a significant change in their dynamics at −40 mmHg compared to baseline and −20 mmHg (Figure 2). This observation leads us to conclude that MAP→RR causality is more sensitive to the early phase of central blood loss simulated by LBNP, thus, a better marker of baroreflex activity when aimed to gain early information regarding progressing blood loss from the circulation. MAP perhaps is a better indicator of early phase of central blood pooling in the lower limbs compared to SBP due its relationship with cardiac output (CO), SVR, and central venous pressure (CVP); *MAP* = (*CO* × *SVR*) + *CVP* (Klabunde, 2011), validation of this hypothesis under gravity-induced orthostatic stress (head-up tilt or stand test) requires future work.

The behavior of non-linear causal heart rate and blood pressure interaction in response to simulated hemorrhage via LBNP is not extensively explored in the literature. The references that exist regarding such behavior have been outlined under orthostatic challenge induced via head-up tilt and stand test, which have considered SPB as a marker of arterial blood pressure. The LBNP is hypothesized to exert orthostatic challenge on the human body analogous to head-up tilt (Taneja et al., 2007; Goswami et al., 2008). Therefore, the physiological response to LBNP is expected to be analogous to that of head-up tilt and quiet standing. Nevertheless, the outcomes of some studies have highlighted the differences in the cardiovascular, cerebrovascular, and the hormonal responses when using LBNP to evoke orthostatic challenge compared to head-up tilt (Hinghhofer-Szalkay et al., 1996; Kitano et al., 2005; Taneja et al., 2007; Bronzwaer et al., 2017).

The absence of gravity induced hydrostatic gradient during lower-body negative suction is shown to be the major contributor toward the existence of such difference. The application of LBNP empties splanchnic blood volume (analogous to hemorrhage) while the gravity induced orthostatic stress (such as, head-up tilt) increases the blood volume in the splanchnic bed (Taneja et al., 2007). Recent work by Silvani et al. (2017) further highlighted such discrepancy, where a significant change in baroreflex response (using SBP) during head-up tilt but not during 1,000 ml of blood loss was observed, similarly, the baroreflex response (SBP→RR) in our analysis failed to differentiate −30 mmHg LBNP (equivalent to approximately 1,000 ml of blood loss) from baseline. These observations, besides highlighting the fact that baroreflex response differs between head-up tilt and central blood loss, raise concern regarding SBP as baroreflex marker when aimed to gain early insights regarding blood loss.

The discrepancies that might exist in the blood pressure regulation via causal heart rate and blood pressure interaction during orthostatic challenge induced by the application of LBNP compared to head-up tilt or stand test is not the scope of this article, and future work shall follow to address such concerns to further our understanding regarding underlying physiology. The current article aimed at investigating the capability of causal heart rate and blood pressure interaction in tracking progressing simulated hemorrhage. In such context, the findings of the study are promising and underscored capability of MAP→RR causality to differentiate moderate category hemorrhage (−30 mmHg LBNP) from resting baseline.

Although a significant (*p* = 0.004) increase in the baroreflex arm (MAP→RR) of the interaction was observed in response to early phase of moderate category hemorrhage (−30 mmHg LBNP; Figure 2), to gain clinical applicability toward early detection of central hypovolemia, it is necessary to outline the behavior of such compensatory mechanism for individual participants. To this end, the MAP→RR causal activity at resting stage (baseline) was compared with that of −30 mmHg for each participant. Figure 4 details the percent increase or decrease in the behavior of MAP→RR causal activity at −30 mmHg compared to rest for all study participants.

In 22 out of 27 participants, there was an increase in the baroreflex causality (MAP→RR), in 2 participants the causality decreased at −30 mmHg, while in three participants the causality did not change (increase/decrease <1%; Figure 4). Additionally, an abrupt decline in the strength of MAP→RR causality in four participants was observed at −40 mmHg compared to −30 mmHg, which could be symptomatic of pre-syncopal feeling or autonomic decompensation. As such, the relationship between the strength of MAP→RR causality and pre-syncopal feeling shall be explored in the future. The percent increase or decrease behavior of non-baroreflex arms (RR→SBP and RR→MAP) were highly variable, moreover, the non-baroreflex causal activities did not achieve statistical significance (*p* > 0.05) in response to LBNP stages. Accordingly, the outcome of the analysis highlights the sensitivity of the baroreflex mediated heart rate control (MAP→RR) toward monitoring progression of central hypovolemia, ascertained by its significant increase in the strength as early as the onset of moderate category hemorrhage (−30 mmHg LBNP).

### Limitations and future directions

Despite the fact that the current study highlighted the potential of quantified compensatory mechanism toward early detection of hemorrhage progression, there exist certain limitations that need to be discussed. (1) In the case of hemorrhage simulated by LBNP, blood volume is not lost from the circulatory system compared to actual hemorrhage but sequestered in the lower periphery of the body, which reduces venous return and therefore, preload. Since the blood is not lost from the circulatory system, inter-subject variability is expected, due to variable tolerance level of individual participants (Hinojosa-Laborde et al., 2014a). (2) In the current study, the respiration and calf electromyography signals were not acquired, therefore the role of respiration and skeletal muscle pump toward facilitating venous return to the heart was not considered (Rowell, 1993; Miller et al., 2005; van Dijk et al., 2005), and it could also have contributed to the variability in the baroreflex causality discussed in Figure 4. Additionally, the respiration is known to affect both the arterial blood pressure and heart rate and should be incorporated in the causality analysis along with heart rate and arterial blood pressure (Faes et al., 2011; Porta et al., 2012). Thus, the generalization of the behavior of baroreflex mediated heart rate elevation in response to blood loss and potential clinical application toward early detection of hemorrhage requires further study.

(3) The degree of blood pooling achieved as a consequence of LBNP was unknown. To accurately quantify the redistribution of blood volume to the peripheral regions, future work could utilize near-infrared spectroscopy (NIRS) in the experimental protocol, analogous to Blaber et al. (2013). The investigation of the strength of SBP→RR and MAP→RR causalities in relation to the degree of blood pooling, quantified via NIRS will shed further light on the effectiveness of SBP→RR or MAP→RR as a choice of baroreflex marker in response to central hypovolemia. (4) The signals considered for quantifying directional interaction are commonly employed vital sign monitors, therefore, clinical application in a hospital setting is feasible. For application in a battlefield, home, or rural settings, a less sophisticated way of blood pressure estimation, e.g., pulse transit time (Mukkamala et al. (2015) should be considered, which would require a sensor placement on the xiphoid process and on a finger (Verma et al., 2015a,b; Yang and Tavassolian, 2017). Feasibility of such system should be investigated in the future.

## Conclusion

The current work studied the compensatory behavior responsible for blood pressure regulation with the progressing LBNP, of which the findings showcase the potential of causal heart rate and blood pressure interaction toward differentiating the severity of simulated hemorrhage. We demonstrated that non-baroreflex causality dominates during resting stage followed by no significant (*p* > 0.05) change in response to the LBNP. On the contrary, the baroreflex causality increased with the progression of LBNP and can be differentiated from rest at the onset of a moderate level of simulated hemorrhage (−30 mmHg LBNP).

Additionally, elevation in the baroreflex arm of the interaction was observed in 82% of the study participants, which further accentuates its potential toward early identification of blood loss. The inter-subject variability in the baroreflex causality in response to progressing hypovolemia due to several physiologic factors such as, tolerance level, presyncope symptom, and autonomic decompensation should be addressed in the future work to improve clinical applicability. Pulse pressure, traditionally considered an early indicator of blood loss was unsuccessful in differentiating progression of central hypovolemia until its advancement to the tail end of the moderate category of hemorrhage (−40 mmHg). Therefore, the compensatory mechanisms shall also be considered in addition to the traditional non-invasive markers of central blood loss (i.e., heart rate and arterial pulse pressure) to gain early and reliable insights regarding the progression of hemorrhage for effective surgical triage.

## Author contributions

AV and KT conceived research. AB and KT designed experiment. AB and KT performed data acquisition. DX and KT pre-processed data. AV analyzed data, performed statistical analysis, created figures and tables, and drafted the manuscript. AV, DX, AG, AC, NG, AB, and KT, interpreted results. All authors read, edited, and approved the final manuscript for publication.

### Conflict of interest statement

The authors declare that the research was conducted in the absence of any commercial or financial relationships that could be construed as a potential conflict of interest.
